# Chronic stress triggers divergent dendritic alterations in immature neurons of the adult hippocampus, depending on their ultimate terminal fields

**DOI:** 10.1038/s41398-019-0477-7

**Published:** 2019-04-26

**Authors:** Chrysoula Dioli, Patrícia Patrício, Nuno Sousa, Nikolaos Kokras, Christina Dalla, Sara Guerreiro, Miguel A. Santos-Silva, Ana Cristina Rego, Luísa Pinto, Elisabete Ferreiro, Ioannis Sotiropoulos

**Affiliations:** 1 Life and Health Sciences Research Institute (ICVS), School of Medicine, University of Minho, Campus de Gualtar, 4710-057 Braga, Portugal; 2 ICVS/3B’s - PT Government Associate Laboratory, 4710-057 Braga/Guimarães, Portugal; 3 First Department of Psychiatry, Eginition Hospital, Medical School, National and Kapodistrian University of Athens, Athens, Greece; 4 Department of Pharmacology, Medical School, National and Kapodistrian University of Athens, Athens, Greece; 5 Center for Neuroscience and Cell Biology (CNC), University of Coimbra, Coimbra, Portugal; 6 Institute of Biochemistry, Faculty of Medicine, University of Coimbra (FMUC), Coimbra, Portugal; 7 Institute for Interdisciplinary Research of the University of Coimbra (IIIUC), Coimbra, Portugal

**Keywords:** Neuroscience, Physiology, Psychiatric disorders

## Abstract

Chronic stress, a suggested precipitant of brain pathologies, such as depression and Alzheimer’s disease, is known to impact on brain plasticity by causing neuronal remodeling as well as neurogenesis suppression in the adult hippocampus. Although many studies show that stressful conditions reduce the number of newborn neurons in the adult dentate gyrus (DG), little is known about whether and how stress impacts on dendritic development and structural maturation of these newborn neurons. We, herein, demonstrate that chronic stress impacts differentially on doublecortin (DCX)-positive immature neurons in distinct phases of maturation. Specifically, the density of the DCX-positive immature neurons whose dendritic tree reaches the inner molecular layer (IML) of DG is reduced in stressed animals, whereas their dendritic complexity is increased. On the contrary, no change on the density of DCX-positive neurons whose dendritic tree extends to the medial/outer molecular layer (M/OML) of the DG is found under stress conditions, whereas the dendritic complexity of these cells is diminished. In addition, DCX+ cells displayed a more complex and longer arbor in the dendritic compartments located in the granular cell layer of the DG under stress conditions; on the contrary, their dendritic segments localized into the M/OML were shorter and less complex. These findings suggest that the neuroplastic effects of chronic stress on dendritic maturation and complexity of DCX+ immature neurons vary based on the different maturation stage of DCX-positive cells and the different DG sublayer, highlighting the complex and dynamic stress-driven neuroplasticity of immature neurons in the adult hippocampus.

## Introduction

One of the most remarkable characteristics of the adult mammalian brain is its ability to adapt to internal and external stimuli, which is reflected in dynamic neuroplasticity phenomena. These neural plasticity processes include changes in synaptic and dendritic morphology, as well as the generation of new cells. Clinical and experimental studies show that brain plasticity is affected by aging^1^, as well as in pathological conditions such as Alzheimer’s disease (AD) and depression^2,3^. Chronic stress, a major precipitant of depression and AD^4–6^ is known to impair brain plasticity, by causing neuronal remodeling related to alterations in dendritic length and arborization^7,8^, as well as by decreasing the number and structure of synapses^9–11^ and newborn cells^12–14^. These alterations affect neuronal function and connectivity with other cells and subsequently the formation and functional dynamics of neuronal circuits and brain networks, leading to impairments in cognitive and affective behaviors^15–18^.

The hippocampus is one of the primary brain areas that show neuronal remodeling under stressful conditions and stress-related brain pathologies, such as AD and depression^12,13,19^. This brain area consists of distinguishable subareas such as the *cornu ammonis* (CA) 1, CA2, CA3 and the dentate gyrus (DG)^20^. Being the input area of the hippocampus, the DG receives projections from the entorhinal cortex (EC) through the perforant pathway while neurons located in the DG project to the pyramidal cells of the CA3^21,22^. In the DG subgranular zone, new neuronal and glial cells are continuously generated throughout life in mammals (including humans) in a process called adult cytogenesis^23,24^. In the final stage of the neurogenic process, immature neurons migrate to the granule cell layer (GCL) where they differentiate into glutamatergic neurons, extending their dendritic tree into the inner and medial/outer molecular layer of the DG (IML and M/OML, respectively) and thus being fully incorporated into the existing network^25^. The dendrites of these newborn neurons form synaptic contacts with axonal projections (perforant pathway) from the EC providing the essential input to the DG and thus, to the whole hippocampus^26–28^.

Converging data support a role for adult hippocampal neurogenesis, namely, in the dorsal region, in certain types of hippocampal-dependent learning and memory, including long-term spatial memory, cognitive flexibility, and pattern separation^29–33^. In brain pathologies characterized by deficits of neuronal plasticity, such as AD and depression, hippocampal neurogenesis was shown to be affected^12,19,34,35^. In line with the suggested role of chronic stress as a risk factor for AD and depression, we have previously shown that chronic stress triggers AD-related cellular mechanisms inducing morphofunctional deficits in (mature) hippocampal neurons, as well as neurogenesis suppression in the DG, leading to cognitive and mood deficits^9,10,13^. Indeed, chronic stress decreases hippocampal neurogenesis in the adult brain by impairing different phases of the neurogenic process^13,36–38^. Despite the plethora of studies showing that chronic stress reduces the number of proliferating cells, as well as immature neurons in the adult hippocampal DG^13,34,39^, there is lack of information about how stress impacts on dendritic development and structural maturation of these newborn neurons and whether immature neurons in different stages of their development are similarly or differentially affected by stress. The latter notion is supported by the fact that the dendritic tree of immature neurons progressively grow into the different DG layers (GCL, IML, M/OML), which are known to exhibit distinct afferents/efferents; thus, growing immature neurons could be exposed to different stimuli during the progressive growth of their dendritic tree. In this study, we monitored how exposure to chronic stress affects structure and complexity of the dendritic tree of doublecortin (DCX)-positive [DCX+] immature neurons in different stages of their development as well as in different layers of the adult DG.

## Materials and methods

### Animals and groups

Wild-type male mice (6–7-month old; C57BL/6J) were used in this study. Mice were housed in groups of 4–5 per cage under standard environmental conditions (8 a.m.–8 p.m. light cycle; 22 °C; 55% humidity, ad libitum access to food and water). Animals were kept and handled in accordance with the guidelines for the care and handling of laboratory animals in the Directive 2010/63/EU of the European Parliament and Council. All experiments were conducted in accordance with the Portuguese national authority for animal experimentation, Direção Geral de Alimentação e Veterinária (ID: DGAV9457). Animals were divided into control and stressed groups (15 animals per group). Stressed animals were exposed to a 9-week chronic unpredictable stress (CUS) paradigm during the daily period of light, consisting of four different stressors: restraint, vibrating platform, overcrowding, and exposure to a hot air stream. Animals were exposed to one stressor per day for 3 h (restraint, vibrating platform, overcrowding) or 30 min (hot air stream). The order of stressors and the time of the day at which the stressor was applied was randomly chosen and varied from week to week to promote unpredictability, as previously described^13,40,41^. During the stress period, control (non-stressed; CON) mice remained undisturbed in their home cages.

### Tissue preparation and immunofluorescence (IF) staining

At the end of the CUS protocol, animals were deeply anesthetized [ketamine hydrochloride (150 mg/kg) plus medetomidine (0.3 mg/kg)] and transcardially perfused with saline followed by ice-cold 4% paraformaldehyde. Brains were removed, post-fixed in 4% paraformaldehyde for 2 h, and then transferred to a 30% sucrose solution until they sunk.

For the morphology analysis of immature neurons, brains were sectioned in a vibratome (Leica, VT1000 S) into 50-μm sections (5 animals per group). Brain sections were double-stained for DCX (for neuronal precursors and immature neurons; 1:500 #sc-8066; Santa Cruz Biotechnology, Dallas, TX, USA) and for postsynaptic density protein 95 (PSD95; 1:2000; Merck Millipore; Darmstadt, Germany), which allows to distinguish the IML and M/OML^42,43^. Briefly, sections were first washed overnight in phosphate-buffered saline (PBS) at 4 °C and further rinsed with PBS at room temperature (RT; 3 × 10 min). Sections were kept in blocking solution of 3% bovine serum albumin (w/v) and 1% Triton X-100 (v/v) in PBS for 1 h at RT. Then, sections were incubated for 3 overnights (4 °C) with the primary antibodies prepared in the blocking solution. After washing in PBS, sections were incubated with the appropriate secondary antibodies (Alexa Fluor 568 donkey anti-goat for DCX, 1:1000, #A11057 and Alexa Fluor 488 donkey anti-mouse for PSD95, 1:1000, #A21202; cell nuclei were stained with Hoechst 33342 (0.2 µg/ml; #H1399, Thermo Fisher Scientific, Waltham, MA, USA). After washing, sections were mounted with Fluorescent Mounting Medium (Fluoroshield Mounting Medium, #ab104135, Abcam).

For GAD67 IF staining, after incubation in sucrose solution, the brains were included in Optimal Cutting Temperature compound (O.C.T.; Tissue Tek, Sakura FineTek, USA), snap-frozen in liquid nitrogen with 2-methylbutane, and then cut in a cryostat into 20-μm sections. These sections were double-stained for glutamic acid decarboxylase (GAD67; for GABAergic synapses; 1:1000, #MAB5406; Merck Millipore; Darmstadt, Germany) and PSD95 (1:400, #75-028; UC Davis/NIH NeuroMab Facility, Davis, CA, USA). Briefly, sections were first washed in PBS (RT, 3 min). For antigen retrieval, sections were heated (15 min in microwave) in citrate buffer (#C9999; Sigma Aldrich/Merck; Darmstadt, Germany). After washing in PBS, cells were permeabilized using PBS-Triton X-100 0.5% (v/v) for 20 min and then, incubated in blocking solution (10% fetal bovine serum (v/v) and 0.5% Triton X-100 (v/v) in PBS (RT, 30 min). After incubation with primary antibodies (4 °C, overnight) and PBS washes, sections were incubated with the appropriate secondary antibodies (RT; 2 h; Alexa Fluor 594 goat anti-mouse for GAD67, 1:1000, #R37121; #A11037; Alexa Fluor 488 goat anti-rabbit, for PSD95; Thermo Fisher Scientific, Waltham, MA, USA). Cell nuclei were stained with 4’,6-diamidino-2-phenylindole (1:1000; Sigma Aldrich) and slides were mounted with PermaFluor Aqueous Mounting Medium (TA-006-FM; Lab Vision, Thermo Fisher Scientific, Waltham, MA, USA). For quantification of the relative fluorescence intensity (FI) of GAD67 staining in different sublayers of DG [GCL, IML, and M/OML], confocal images (×40) of dorsal DG (suprapyramidal blade) were obtained by the confocal microscope Olympus FluoView FV1000 (Olympus, Hamburg, Germany) with the image acquisition parameters being identical for all images. Quantification of FI was performed using the ImageJ software (http://rsb.info.nih.gov/ij/). Briefly, TIFF exported images were converted to RGB stack images and the color channels corresponding to each staining were split. The different sublayers of the DG were selected in the GAD67 channel using the polygon selection tool. PSD95 staining was used as a reference for IML identification. Eight-to-ten images per animal (four animals per group) were used for this analysis and FI of each sublayer was normalized by the corresponding area. The experimenter was blind to the animal’s group.

### Cell density and three-dimensional (3D) reconstruction of DCX+ immature neurons

**Fig. 1:**
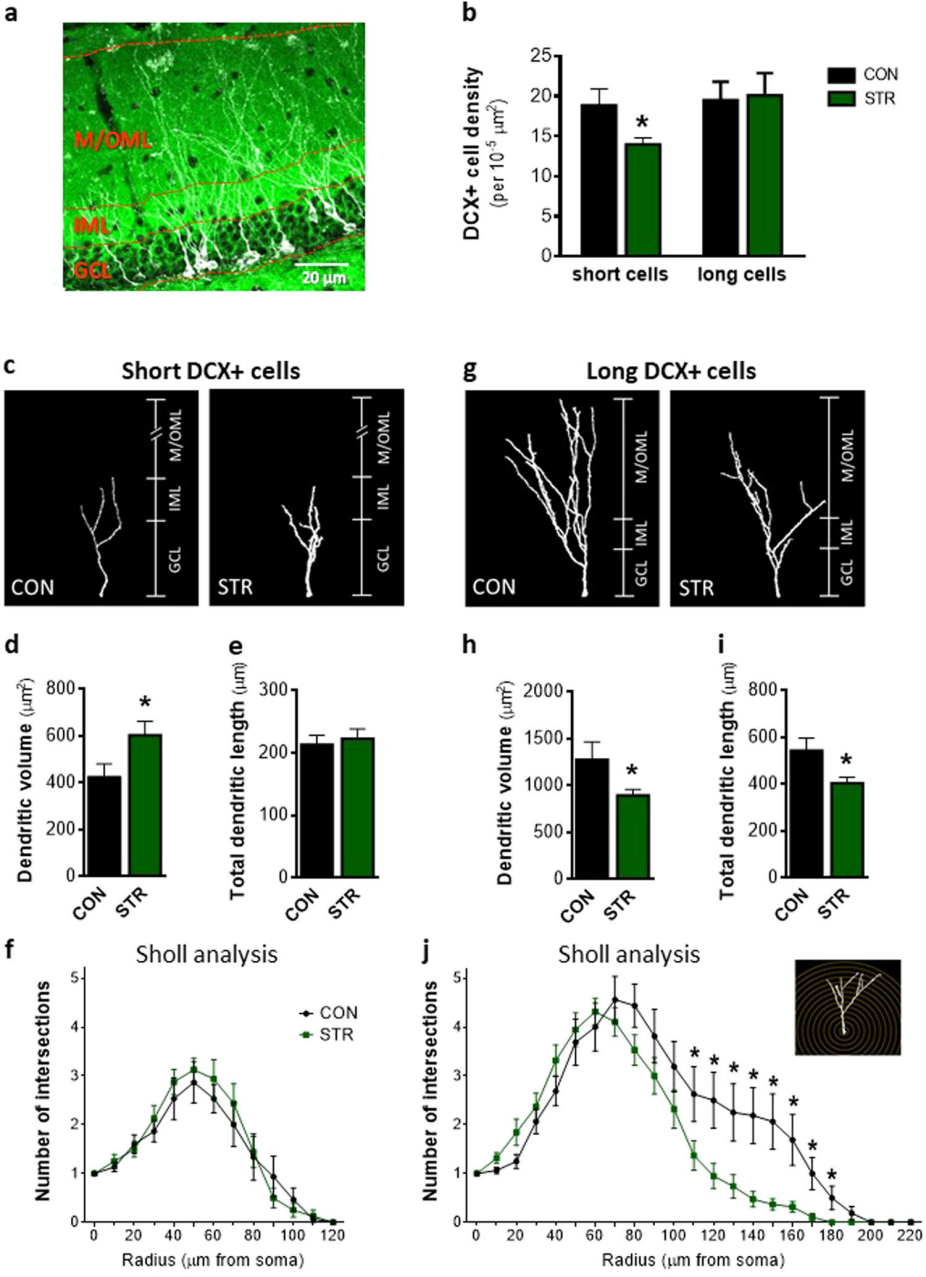
Differential impact of chronic stress on dendritic complexity of immature neurons that reach or not the outer molecular layer (OML) of the adult dentate gyrus (DG). **a** Representative microphoto of doublecortin (DCX) and postsynaptic density-95 (PSD95) staining in the DG of adult mouse showing the granular cell layer (GCL) as well as inner and medial/outer molecular layers (IML and M/OML, respectively) of DG. **b** Chronic stress decreases the density of DCX+ cells (immature neurons) whose dendritic tree reaches the IML (named as short DCX+ cells), whereas it does not affect the DCX+ cells whose dendritic tree reaches the M/OML (namely, long DCX+ cells). **c** Illustration of three-dimensional (3D) reconstruction of short DCX+ cells under control (CON) and stress (STR) conditions. **d**, **e** Short DCX+ immature neurons exhibited higher dendritic volume in stressed animals in comparison to controls without significant alteration of their total dendritic length. **f** Sholl analysis of short DCX+ cells revealed no differences of intersections number between control and stressed DCX+ immature neurons. **g** Representation of 3D reconstruction of long DCX+ cells in the DG of control and stressed animals. **h**, **i** In contrast to short DCX+ cells, the dendritic volume and total dendritic length of long DCX+ immature neurons is lower in stressed animals when compared to controls. **j** Sholl analysis of long DCX+ cells showed reduced number of intersections at the distal part of long DCX+ cells of stressed animals. All numerical data are shown as mean ± S.E.M. (*p < 0.05)

For DCX+ cell density, images were obtained using a Zeiss Axio Observer Z1 widefield microscope equipped with a Plan-ApoChromat ×20/0.8 NA objective and the area of the corresponding GCL was calculated by using the open-access Fiji software^44^. For 3D dendritic reconstruction, confocal stack images were obtained using a confocal microscope equipped with a Plan-ApoChromat ×40/1.4 NA oil-immersion objective and a ×0.7 digital zoom and analyzed by the Simple neurite tracer plugin^44,45^ using the open-access Fiji software, as previously described^42^. PSD95 staining was used for layer separation between IML and M/OML (see Fig. 1a). In this study, only DCX+ cells that branch into the GCL and reach the molecular layer (ML) were analyzed. Sholl analysis was performed using the “3DSholl analysis” plugin (http://fiji.sc/Sholl_Analysis) and based on the quantification of the number of intersections between dendrites and the surface of spheres with a radius increment of 10 μm. Dendrite length and complexity was further analyzed with the Skeletonize3D (https://imagej.net/Skeletonize3D) and AnalyzeSkeleton (https://imagej.net/AnalyzeSkeleton) plugins. The experimenter was blind to the animal’s group.

### High-performance liquid chromatography (HPLC) analysis

The analytical measurements were performed using a GBC LC1150 (GBC Inc, Braeside, Australia) HPLC pump coupled with a BAS LC4C (Bioanalytical Systems, West Lafayette, IN, USA) electrochemical detector and pre-column derivatization, as previously described^46,47^ with some minor modifications. The column used was Hypersil Gold aQ, 150 mm × 2.1 mm^2^, 5 μm (Thermo Fisher Scientific, MA, USA). The voltage of the working electrode was set at +800 mV. The mobile phase consisted of an 4:96 water:acetonitrile (Chem-Lab, Belgium) buffer containing 100 mM monosodium phosphate and 0.5 mM Na_2_EDTA (AppliChem, Germany), regulated at pH = 5.5. All samples (8 animals per group) were diluted 1:100 with ddH_2_O, then further diluted 1:1 with 0.1 M Borax buffer (Sigma–Aldrich, St. Louis, USA) containing o-phthalaldehyde (Sigma–Aldrich) regulated at pH 10.4 and they were left at RT for 10 min prior to injection. Quantification of glutamate, GABA, and aspartate was done by comparison of the area under the curve with that of reference external standards using the chromatography software (Clarity, Data-Apex, Czech Republic)^48^.

### Statistical analysis

Data were analyzed using GraphPad Prism v.6.01 (GraphPad Software, La Jolla, CA, USA). Student’s *t* test analysis was applied whenever two groups were compared. For Sholl analysis, mixed-design analysis of variance (ANOVA) repeated measurements was used. Differences were considered statistically significant when *p* < 0.05. Results are presented as mean ± S.E.M.

## Results

### Chronic stress impacts differently on immature neurons reaching the IML or M/OML of the adult dorsal DG

To clarify the impact of chronic stress on dendritic development and structural maturation of immature neurons in the adult dorsal DG, we exposed mice to a 9-week CUS paradigm and monitored different parameters of dendritic structure and complexity of immature neurons based on DCX staining. DCX is a cytoskeletal protein expressed both in neuronal progenitors and immature neurons^49,50^. We monitored DCX+ cells in distinct phases of maturation that branched into the GCL and reached the ML while the DG sublayers, namely, GCL, IML, and M/OML were used as the regions of interest for layer-differential monitoring of dendritic complexity. Among DCX+ immature neurons, only those reaching the M/OML participate directly in the DG connectivity with the EC. Thus we divided the DCX+ immature neurons into: (a) DCX+ cells that exhibit dendrites branching to the IML but do not reach to the M/OML (short DCX+ cells; Fig. 1a, c) and (b) DCX+ cells with dendritic tree reaching to the M/OML (long DCX+ cells; Fig. 1a, g).

As shown in Fig. 1b, exposure to chronic stress resulted in decreased density of short DCX+ cells (*p* = 0.035), while no differences were observed in the density of long DCX+ cells. Similarly, the percentage of short, but not long, DCX+ cells was reduced by stress [for short DCX+ cells, CON: 34.03 ± 2.216, STR: 24.95 ± 1.262; *p* = 0.002; for long DCX+ cells, CON: 32.42 ± 1.536, STR: 33.96 ± 2.286]. Additionally, 3D reconstruction of the dendritic arborization of both short and long DCX+ cells was performed. As shown in Fig. 1c, d, short DCX+ cells exhibit increased dendritic volume in the DG under stressful conditions (*p* = 0.03), when compared to cells of control conditions (Fig. 1d), without significant change of their total dendritic length (Fig. 1e). Moreover, we performed Sholl analysis, an additional structural measure reflecting changes of dendritic complexity in different dendritic parts based on their distance from the soma. Mixed-design statistical analysis showed no impact of chronic stress in the number of dendritic intersections at various distances from the soma of short DCX+ cells (Fig. 1f). Interestingly, long DCX+ cells of the DG exhibited a completely different profile under stress conditions. We found that the dendritic volume of long DCX+ cells in stressed animals was diminished when compared to control ones (*p* = 0.04; Fig. 1g, h). Similarly, long DCX+ cells under stressful conditions exhibited lower total dendritic length of the long DCX+ cells in comparison to control animals (*p* = 0.02) (Fig. 1i). Mixed-design ANOVA statistical analysis of data obtained by Sholl analysis revealed a significant within-subject effect of radius (distance from soma) (*F*_4.40, 145.28_ = 41.93, *p* < 0.001). There was a significant between-subjects effect of group (*F*_1,33_ = 4.82, *p* = 0.035) with cells of stressed animals displaying overall reduced dendritic intersections (mean = 1.81, SE = 0.185 and mean = 2.41, SE = 0.201 for stressed and control animals, respectively). Moreover, there was a significant *Stress* × *Distance* interaction (*F*_4.40, 145.28_ = 23.21, *p* = 0.005) with long DCX+ cells of stressed animals exhibiting lower number of intersections at a distance from 110 to 180 μm (*p* = 0.040; *p* = 0.010; *p* = 0.010; *p* = 0.003; *p* = 0.003; *p* = 0.008; *p* = 0.007; *p* = 0.030, respectively, for each 10 μm for 110–180) when compared to control ones (Fig. 1j). The above results suggest a differential response of the short and long DCX+ immature neurons to chronic stress.

### Compartmentalized effect of chronic stress on structural complexity of short DCX+ immature cells in the adult DG

**Fig. 2:**
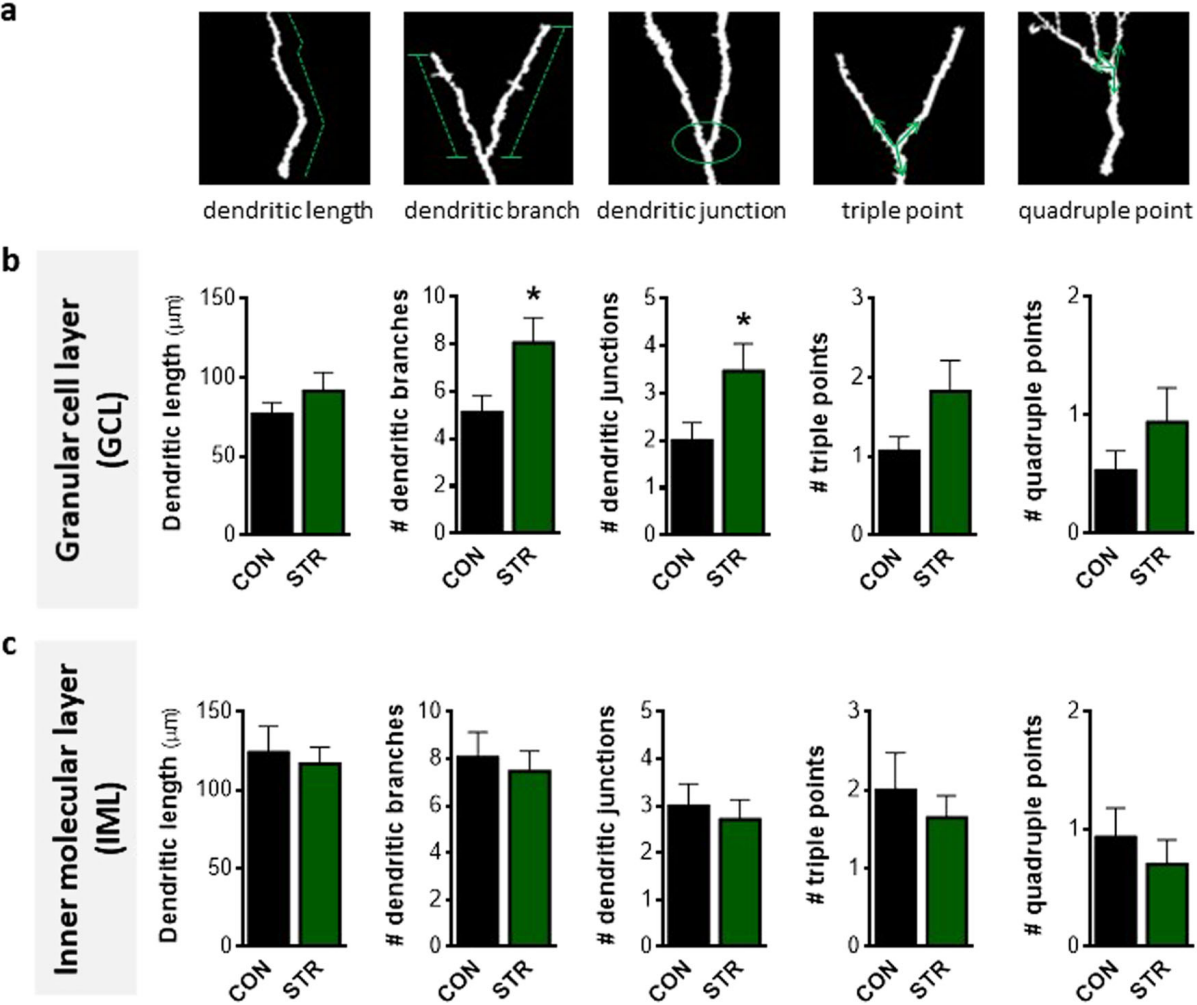
Chronic stress increases structural complexity of short doublecortin-positive (DCX+), immature neurons affecting granular cell layer in the adult dentate gyrus. **a** Three-dimensional-based illustration of different parameters of dendritic complexity, such as dendritic length, dendritic branches, dendritic junction, and triple and quadruple points, monitored in this study. **b** Short DCX+ immature neurons of chronically stressed animals exhibit increased number of branches and junctions in the granular cell layer (GCL) dendritic compartment when compared to control animals; however, no significant stress-driven effects are found in dendritic length and the number of triple and quadruple points of the short DCX+ immature neurons. **c** No differences were found in all structural parameters of the short DCX+ dendritic compartment allocated in the inner molecular layer (IML) after exposure to chronic stress. All numerical data are shown as mean ± S.E.M. (*p < 0.05) CON control, STR stressed

Based on the above-described findings supporting a differential stress response of DCX+ cells whose dendritic tree reaches to the IML or the M/OML of the DG, we next monitored various structural parameters of dendrites such as dendritic length, branches, junctions as well as triple and quadruple points (Fig. 2a) of both short and long DCX+ cells in different DG sublayers, namely, the GCL, IML, and M/OML. Our analysis showed that short DCX+ cells exhibited increased number of dendritic branches (*p* = 0.033; Fig. 2b) and dendritic junctions (*p* = 0.046; Fig. 2b) in the GCL in stressed animals when compared to controls. While a similar trend was found for dendritic length and the number of triple and quadruple junction points, these differences were not significant (Fig. 2b). In contrast to the GCL, chronic stress did not affect any of the above-mentioned structural parameters of short DCX+ cells in the IML, as monitored by 3D-reconstruction analysis (Fig. 2c).

### Chronic stress impacts differently on the structural complexity of immature neuron dendritic compartments localized in the GCL and M/OML

**Fig. 3:**
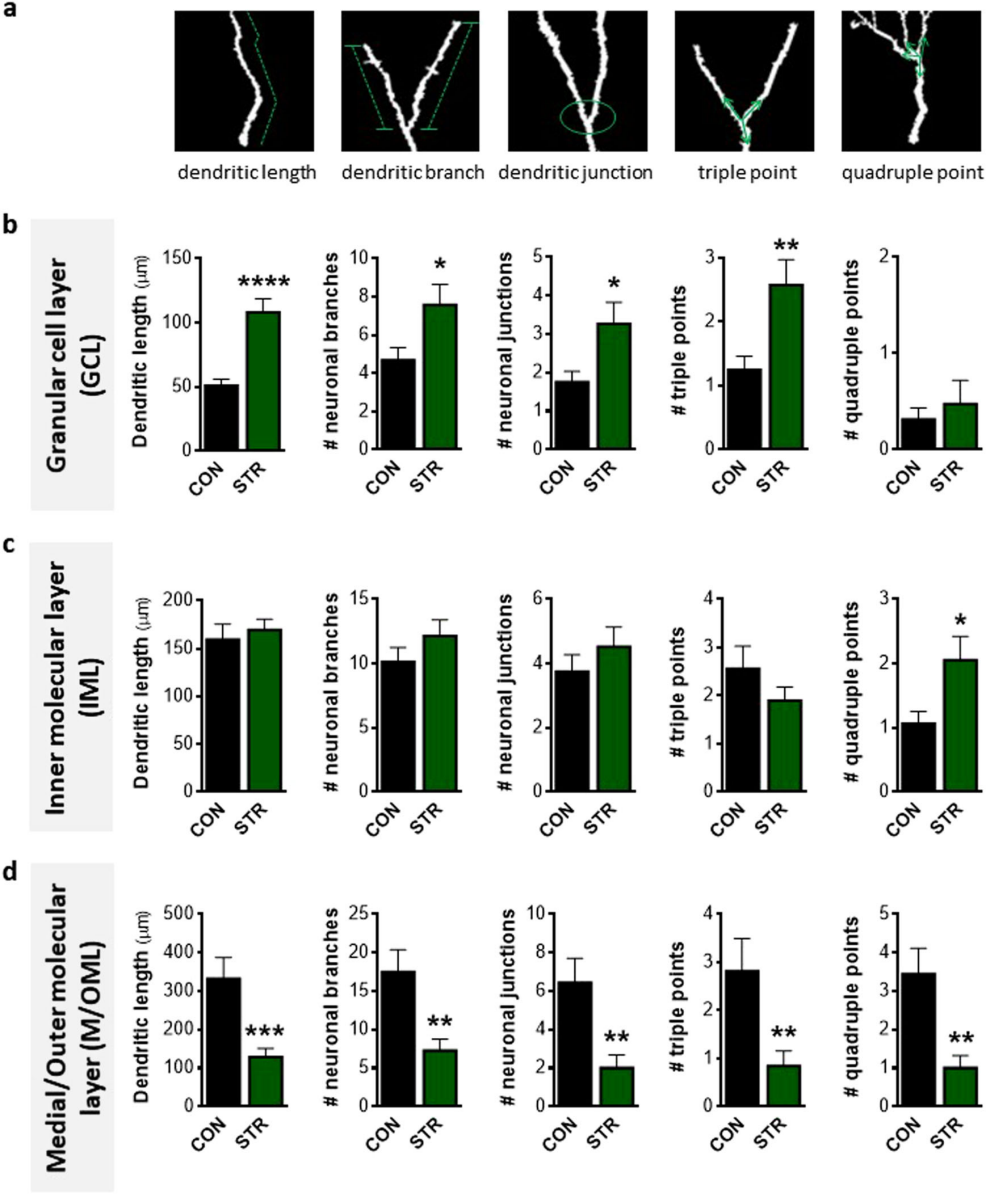
Long doublecortin-positive (DCX+) immature neuron structural complexity is differentially affected by chronic stress in distinct dendritic compartments located into the granular cell and medial/outer molecular layers (GCL and M/OML, respectively) of the adult dentate gyrus. **a** Three-dimensional-based illustration of different parameters of dendritic complexity (dendritic length, dendritic branches, dendritic junction, triple and quadruple points). **b** Dendritic length, number of branches, junctions, and triple points of long DCX+ cells are higher in the dendritic compartment found at the GCL of the stressed animals in comparison to controls. **c** In contrast to the GCL, we detect no differences in dendritic length, number of branches, junctions, and triple points in the IML dendritic compartment besides a stress-driven small, but significant, increase in the number of quadruple points. **d** Length of M/OML dendritic compartment of long DCX+ cells is lower in stressed animals when compared to controls followed by reduced number of branches, junctions, triple points, and quadruple points. All numerical data are shown as mean ± S.E.M. (*p < 0.05; **p < 0.01; ***p < 0.001; ****p < 0.0001) CON control, STR stressed

We next analyzed the dendritic tree of long DCX+ cells along the different DG sublayers. Similar to the short DCX+ cells, the dendritic part of the long DCX+ cells located in the GCL of the stressed animals displayed a more complex and longer arbor when exposed to chronic stress. Specifically, long DCX+ cells of stressed animals exhibited increased dendritic length (*p* < 0.001; Fig. 3b), the number of dendritic branches (*p* = 0.035; Fig. 3b), and junctions (*p* = 0.030; Fig. 3b), as well as the number of triple points (*p* = 0.009; Fig. 3b) of dendritic compartments inside the GCL, in comparison to control animals; no impact of chronic stress was observed in the number of quadruple points in the GCL (Fig. 3b). In line to short DCX+ cells, no effects of chronic stress were found in these structural parameters of long DCX+ cells in the IML, besides a stress-driven increase in quadruple junctions (*p* = 0.031; Fig. 3c).

Interestingly, the stress effect on the structural complexity of long DCX+ cells in the M/OML was completely opposite to the one in the GCL. The M/OML dendritic compartment of the long DCX+ cells in the stressed animals was shorter (*p* < 0.001) compared to control animals (Fig. 3d). Similarly, the number of M/OML dendritic branches (*p* = 0.002) and junctions (*p* = 0.003) as well as the number of triple and quadruple junction points (*p* = 0.009 and *p* = 0.001, respectively) of long DCX+ cells was also lower in the stressed animals when compared to controls (Fig. 3d]. Overall, the above data demonstrated for the first time that long DCX+ cell dendritic compartments are differentially affected by chronic stress depending on the DG sublayer where these dendrites are located.

### Chronic stress impacts GABAergic neurotransmission in the GCL of adult DG

**Fig. 4:**
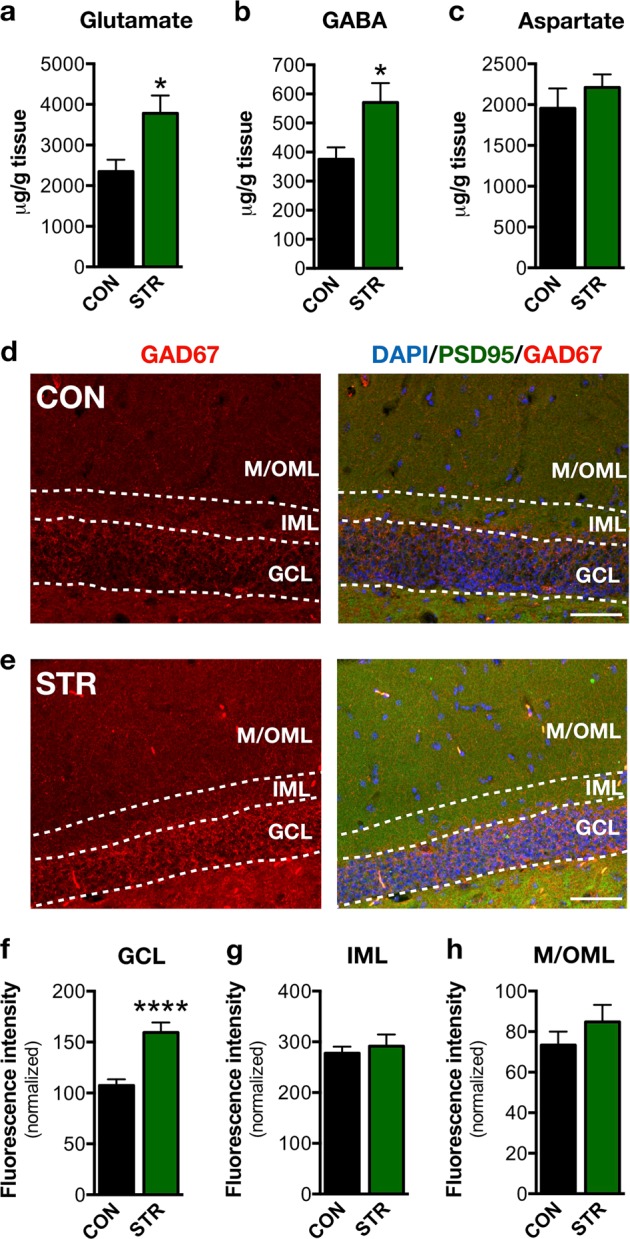
Impact of chronic stress on GABAergic neurotransmission in adult dentate gyrus (DG). **a**–**c** High-performance liquid chromatographic (HPLC) analysis showed that glutamate and GABA levels are increased in the dorsal hippocampal part of the stress-exposed animals; note that aspartate levels are not altered after chronic stress exposure. **d**, **e** Representative microphoto of the GAD67 and PSD95 immunostaining in the DG of control and chronically stressed mice. Scale bar represents 50 µm. **f**–**h** Chronic stress increased the GAD67 fluorescence intensity selectively in the granular cell layer of the DG without affecting GAD67 in the inner molecular layer and medial/outer molecular layer sublayers. All numerical data are shown as mean ± S.E.M. (*p < 0.05; ****p < 0.0001); CON control, STR stressed

Based on the suggested role of γ-amino butyric acid (GABA) on promoting dendritic complexity in the GCL of the DG^51^ and the hypothesized contribution of the imbalance between GABAergic and glutamatergic transmission in damaged adult neurogenesis, we assessed the levels of GABA, its precursor neurotransmitter, glutamate, as well as the enzyme glutamic acid decarboxylase (GAD) that converts glutamate to GABA. HPLC analysis of the dorsal hippocampus of control and stressed mice revealed that both glutamate and GABA levels were increased under stress conditions (*p* = 0.016 and *p* = 0.025, respectively; Fig. 4a, b). We also analyzed the levels of aspartate in the hippocampus finding no significant differences between control and stressed animals (Fig. 4c). We next performed IF staining for GAD67, which catalyzes the decarboxylation of glutamate to GABA for neuron activities related to synaptogenesis and protection from neural injury^52^. While GAD67 staining was detected in all the sublayers of the DG, chronically stressed animals exhibited a selective increase of GAD67 fluorescence intensity levels in the GCL of the DG (*p* < 0.0001; Fig. 4d–f), while no stress-driven differences were found in other DG sublayers, namely, IML and M/OML (Fig. 4d, e, g, h).

## Discussion

An important hallmark of adult hippocampal neural plasticity is its involvement in diverse stimuli and brain pathologies^27,53,54^. Clinical and experimental evidence supports a detrimental and cumulative impact of prolonged stress on the precipitation of brain pathologies such as AD and depression^6,34,55,56^ through the well-established role of chronic stress as a negative regulator of hippocampal neuroplasticity. Exposure to chronic stress is shown to impact on dendritic and synaptic remodeling and decrease cell genesis^7,12,57,58^. However, its role as a neuronal remodeler during early stages of neuronal maturation in the adult brain has not been addressed previously. Here we have focused on the effects of chronic stress on immature neurons of the dorsal hippocampal DG during distinct phases of their maturation; the DG is a region where new neurons have been proposed to contribute to the learning and formation of new memories^28^.

**Fig. 5:**
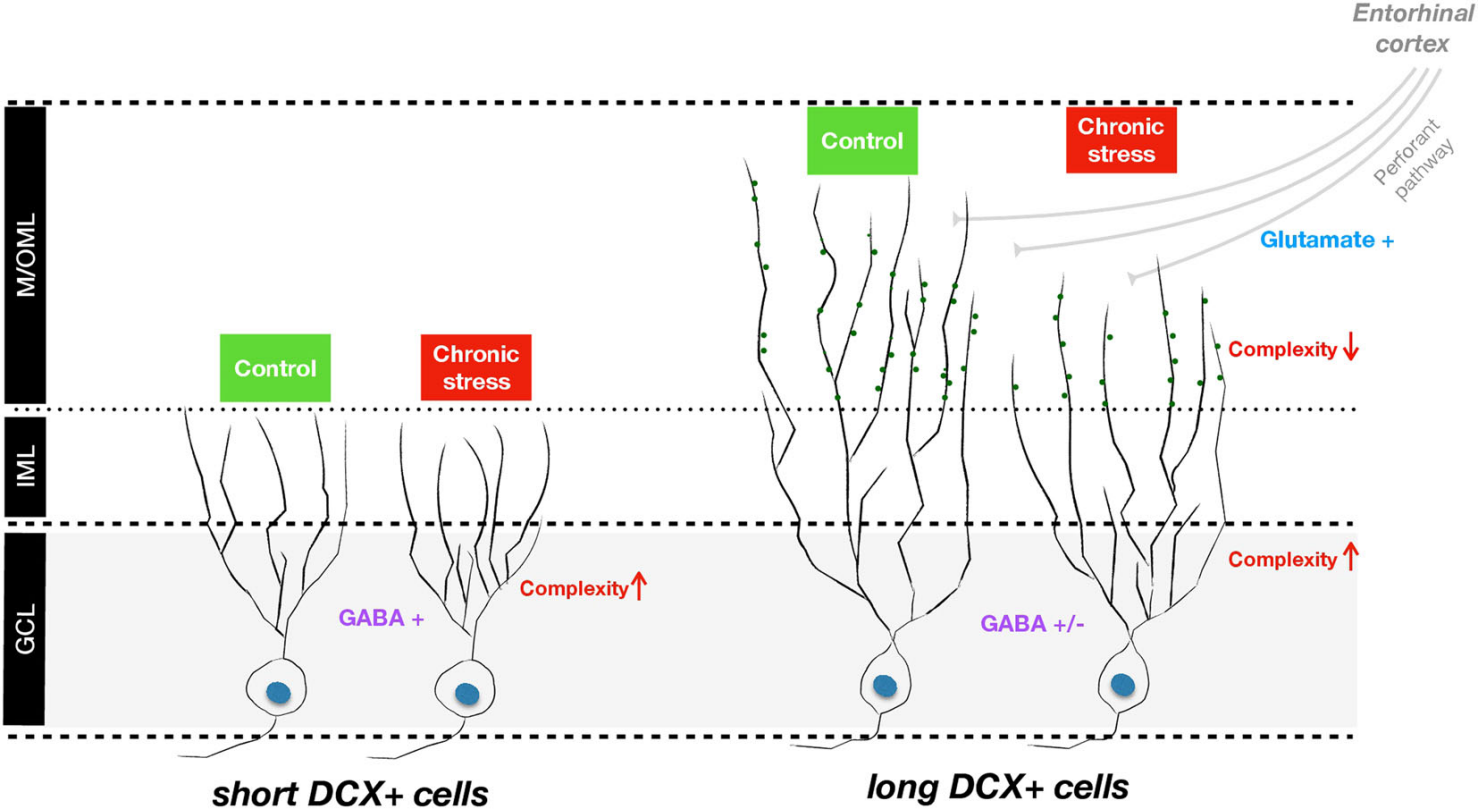
Working model summarizing the differential neuroplastic effects of chronic stress over the doublecortin-positive (DCX+) hippocampal immature neurons. DCX+ immature neurons whose dendrites reach solely the inner molecular layer (short DCX+ cells) and DCX+ immature neurons that reach the medial/outer molecular layer (M/OML; long DCX+ cells). In both short and long DCX+ cells, chronic stress promotes complexity of the dendrites located on the granular cell layer possibly due to the stress-driven increase in GABA release and transmission in this region (Hu et al.^65^); note that GABA is shown to promote dendritic growth and arborization in dentate gyrus^51^. On the other hand, the M/OML dendritic compartment of long DCX+ cells, which start receiving entorhinal cortex (EC) glutamatergic projections, exhibit reduced complexity under stress conditions, an effect that may result from impaired glutamatergic neurotransmission due to chronic stress

A widely used marker of newly born immature neurons in the adult brain is DCX, a cytoskeletal protein that is transiently expressed from an early stage of progenitor cell differentiation along the neuronal pathway; peak DCX expression is seen at around 10 days after cell division, whereafter it decreases to negligible amounts at day 30^59,60^. During this period, the neuronal dendritic tree grows progressively into the MLs of the DG, allowing the identification of a cell’s maturational stage according to particular morphological features^61^. For instance, newborn hippocampal granule cells extend spineless apical dendrites that reach the IML, but not the M/OML, at around 10 days after birth^62^. Later, aged at around 14–21 days, while still expressing DCX, newborn DG cells begin display dendritic branching and reach the M/OML^62^. Although DCX+ newborn cells are heterogeneous (aged between 0 and 30 day), our analysis focused on the DCX+ cells that exhibit a dendritic tree entering the MLs, by monitoring the dendritic plasticity and maturation of DCX+ immature neurons under stressful conditions (see Fig. 5). Considering this, we subdivided the DCX+ population of interest in distinct phases of maturation based on the cells’ dendritic extension solely into the IML (short DCX+ cells) or both IML and M/OML (long DCX+ cells).

Many experimental studies show that DG newborn neurons become atrophic after prolonged exposure to stressful conditions. However, the current study demonstrates, for the first time, that dendritic length and complexity (as assessed by 3D-reconstruction and Sholl analysis of dendritic trees) are differentially affected by chronic stress, with only a subpopulation of DCX+ immature neurons’ dendrites reaching the M/OML of the DG. In contrast to the lower dendritic volume and shorter length of long DCX+ cells from stressed animals (see Fig. 1), short DCX+ cells exhibit increased dendritic volume and increased dendritic branches and junctions in the GCL (see Figs. 1 and 2). We cannot exclude the possibility that the increased dendritic volume and complexity observed in the short DCX+ cells after stress exposure represents a compensatory mechanism to counterbalance the reduced number of short DCX+ cells, i.e., a plasticity mechanism to (partly) reinstate local neuronal connectivity. Moreover, within the first few days after birth (while located inside the GCL), newborn cells start receiving synaptic GABAergic input, presumably from local interneurons in the GCL, but not glutamatergic inputs, consistent with the absence of dendritic spines in the ML at this time point^28,63,64^. Note that GABAergic input to these cells is excitatory (rather than inhibitory) and is suggested to promote their survival and maturation. A previous study also demonstrated that specific (local) administration of a GABA receptor agonist in the DG promotes dendritic growth and arborization in dentate granule cells^51^ and exposure to stress is suggested to trigger hippocampal GABAergic transmission^65–67^. In line with these findings, we show that exposure to chronic stress increases hippocampal GABA levels, a phenomenon accompanied by a selective increase of GAD67 in the GCL (but not IML or M/OML) of the DG, indicative of increased GABAergic neurotransmission in this layer. GAD67 is an enzyme that catalyzes the decarboxylation of glutamate to GABA for neuron activities related to synaptogenesis and protection from neural injury^52^. As GABA in the DG is suggested to promote dendritic complexity^51^, this could serve as a plausible explanation for the increased dendritic complexity in the GCL of the adult DG under stress conditions. It remains to be clarified whether this selective GCL-localized increase of dendritic complexity of the DCX+ cells may be related to locally restricted GABAergic innervation on the GCL and whether this dendritic response to GABA is limited to this developmental stage. In fact, short DCX+ cells dendrites lie mainly inside the GCL and do not reach the M/OML and they are most likely aged <14 days; here it deserves mentioning that previous studies suggest that immature neurons aged 7–14 days only receive GABA excitatory inputs^62^. Notably, the complexity in the GCL dendritic compartment was also higher under stress conditions in long DCX+ immature neurons, the other DCX+ cell subpopulation monitored in this study (see Figs. 1 and 3). This could be attributed to the fact that stress leaves a morphological “scar” on short DCX+ cells that is still observed at later stages of their development and maturation (long DCX+ cells). It is noteworthy that 9-weeks stress used in these studies is long enough to affect the entire period of DCX+ cell maturation as DCX is expressed until around day 30 after cellular birth.

The long DCX+ cells represent more mature newborn neurons, as these DCX-expressing newborn cells begin to show dendritic branches that reach the M/OML at around 14 days of age^62^. This is accompanied by the development of spines and overlaps with the transition of GABA from a depolarizing to hyperpolarizing mode and the glutamatergic inputs to these neurons (14–21 days; see also Fig. 5)^62^. In contrast to the stress-driven increased complexity of the GCL dendritic compartment, dendrites extending into the M/OML of the DG are significantly shorter and less complex in stressed animals; these observations points toward a DG sublayer-specific neuroplastic response of immature neurons to chronic stress. The reduced length and complexity of the dendritic compartment located in the M/OML of the (long) DCX+ cells in stressed animals could be attributed to either diminished dendritic elongation of the developing dendritic tree inside the M/OML or retraction/simplification of existed M/OML dendritic tree of the long DCX+ immature neurons triggered by chronic stress. Importantly, the M/OML, but not GCL, of the DG is the sublayer receiving glutamatergic input from entorhinal cortical neurons (perforant pathway; see Fig. 5). Whereas glutamatergic neurotransmission at this stage is recognizably relevant for the development and survival of newborn neurons^68^, its disruption in the context of stress may trigger significant neuronal impairments. In line with our finding of a stress-evoked increase of glutamate levels, previous studies have shown that chronic stress and the main stress hormones, glucocorticoids affect glutamatergic transmission, including increase on glutamate release, changes in glutamate receptors, and glutamate metabolism/clearance, with major implications for morphology and survival of adult neurons^69–71^. Although the mechanisms through which chronic stress impacts on glutamate release have not been widely investigated, previous studies suggest increased neuronal presynaptic glutamate uptake and glutamate release^70^, as well as upregulation of the glia glutamate transporter (GLT-1) expression^71^. An additional mechanistic explanation for the reduced structural complexity of the M/OML dendritic compartment in DCX+ immature neurons under stress may include the previously described stress-driven reduction of neurotrophic factors, such as brain-derived neurotrophic factor (BDNF)^72^. BDNF has a primarily autocrine action in the promotion of dendrite morphogenesis^73,74^ and is strongly expressed in mature and newly generated granule neurons, where it is anterogradely transported to mossy fibers^75,76^. Indeed, many studies have connected reduced BDNF levels and deficits in hippocampal neurogenesis with cognitive deficits in chronically stressed animals^77^. While beyond the focus of the current work, previous studies have associated reduced neurogenesis with specific DG-dependent cognitive deficits such as pattern separation^13,33,78^. Further studies should focus on the understanding and dissection of the contribution of immature neurons to stress-induced DG-dependent memory impairment as well as the role of high glucocorticoid levels on DG cells at differing states of maturity (proliferating cells, neuroblasts, newborn astrocytes, and newborn neurons) since glucocorticoids are known to suppress neuronal birth and to influence neuronal fate^79,80^.

The differential connectivity of the IML vs. M/OML may also account for the different impact of chronic stress in various dendritic compartments of immature DCX+ cells. In fact, afferents to the DG come from different sources and areas, and whereas the primary cortical input to the DG is the glutamatergic projection from layer II of the EC, there are other fundamental differences between IML and OML regarding neuronal projections. For instance, the lateral EC, which integrates novel environmental information, and the medial EC containing grid cells with spatial specificity innervate the OML and the MML, respectively^81^. Moreover, mossy cell axons are the major afferent input to the IML. The IML also receives inputs from supramammillary, cholinergic, mossy cell, and ventral CA3 pyramidal cell axons^81,82^. Future studies should further dissect the dynamic connectivity of the DG with the rest of the hippocampus and the cortex under control and stressful conditions.

We acknowledge that inclusion of female animals would provide extra and essential information about the potential role of sex on the effects of stress on DG neuron maturation. Indeed, a plethora of previous studies have demonstrated sex differences in the susceptibility to stress as well as hippocampal regulation of the stress response^48,83,84^ and such studies are therefore currently being undertaken. For example, exposure to acute stress is known to oppositely affect spine density in the CA1 area of the hippocampus in male and female animals^85^. In addition, sex hormone fluctuations during the estrous cycle of females induce oscillations in the levels of proliferation and in the maturation of DG hippocampal newly born neurons^86^. Moreover, exposure to high levels of glucocorticoids and chronic uncontrollable footshock stress decreases hippocampal proliferation in male but not in female rodents^87,88^.

In summary, the current study suggests that the effect of chronic stress on the development and maturation of the dendritic tree of DG immature neurons in the adult brain may vary depending on their distinct maturation stage as well as the specific sublayer of the DG within which their dendritic compartments are localized (see Fig. 5). Future studies should clarify whether spine changes are involved in the differential effects of chronic stress on the structural profile DG neurons at different stages of maturation, aiming to understand that the underlying mechanisms of the divergent dendritic alterations in immature hippocampal neurons depend on their ultimate terminal fields. A better understanding of the mechanisms underpinning the effects of chronic stress throughout the span of adult neurogenesis may help the development of targeted therapies for stress-related disorders.
